# Resilience of Norovirus GII.4 to Freezing and Thawing: Implications for Virus Infectivity

**DOI:** 10.1007/s12560-012-9089-6

**Published:** 2012-10-12

**Authors:** Gary P. Richards, Michael A. Watson, Gloria K. Meade, Gregory L. Hovan, David H. Kingsley

**Affiliations:** 1 United States Department of Agriculture, Agricultural Research Service, Delaware State University, James W.W. Baker Center, Dover, DE 19901 USA; 2 Delaware Division of Public Health Laboratory, Smyrna, DE 19977 USA

**Keywords:** Norovirus, Freezing, Thawing, Persistence, Infectivity, Capsid integrity

## Abstract

Genogroup II.4 norovirus (NoV) remains the predominant NoV strain in food- and water-borne outbreaks. Capsid integrity as well as viral RNA persistence were determined for GII.4 NoV by real-time RT-PCR after 1–14 freeze/thaw (F/T) cycles (−80 °C/+22 °C) or after −80 °C storage for up to 120 days. In both cases, capsid integrity and viral RNA titers remained stable. RNase was exogenously added after 1–14 F/T cycles, but did not alter the amount of genomic NoV RNA detected, indicating that capsids remained intact. Presumptive NoV infectivity was evaluated in functional studies by a porcine gastric mucin binding assay. Viruses frozen and thawed up to 14× bound similarly to porcine mucin, suggesting no reduction in virus infectivity. Overall, this study shows that a) NoV particles retain their integrity for at least 14 F/T cycles, b) long-term (120 day) frozen storage does not decrease NoV RNA titers, and c) capsid binding to receptor-like glycoprotein moieties remains unaltered after 14 F/T cycles. This work indicates that freezing and thawing of foods or beverages would not be a practical processing intervention to reduce NoV contamination. Likewise, repeated freezing and thawing, as might be encountered during winter months, is not expected to inactivate NoV in the environment. Results do show that laboratory samples destined for molecular biological analyses or for use as positive controls may be repeatedly frozen and thawed without any anticipated reduction in NoV RNA titers. This study documents the cryostability of NoV capsids and RNA to freezing and thawing and to the possible retention of virus infectivity.

## Introduction

Human norovirus (NoV) is the principle cause of food-borne illness in the United States with an incidence recently estimated at 5.5 million cases annually (Scallan et al. 2011). Water-borne illnesses from NoV are estimated to be even higher than food-borne illnesses (Mead et al. 1999). Genogroup II clade 4 (GII.4) NoV is the most prevalent strain worldwide accounting for about 80 % of illnesses (Donaldson et al. 2008; Siebenga et al. 2007, 2009; Hall et al. 2011). Noroviruses contain single stranded, positive sense, RNA genomes of 7.7 kb surrounded by an icosahedral capsid 27–40 nm in diameter. Reverse-transcription-PCR remains the only practical method to detect human strains of NoV in food, environmental, and clinical samples since these viruses cannot be propagated in traditional cell culture systems or in animal models. The RNA genome of NoV is normally protected by a resilient capsid, but damage to the capsid’s integrity makes the RNA molecule highly susceptible to degradation by ubiquitous RNases in the environment. Thus, the integrity of the capsid is fundamental to the integrity of viral RNA.

Only a limited number of studies have assessed the effects of freezing duration or freezing and thawing on human NoV. Such studies have practical application in identifying: a) potential food processing techniques to eliminate NoV in foods and beverages, b) environmental conditions conducive to the inactivation of NoV, and c) methods for the preservation of viruses for use in NoV testing laboratories. Butot et al. (2008) showed minimal losses in feline calicivirus titers in berries and herbs after 90 days of frozen storage with more acidic foods causing greater decreases. Feline caliciviruses are a commonly used surrogate for human NoV. Mormann et al. (2010) evaluated GII.3 NoV RNA reduction after various processing treatments in experimentally contaminated foods. They showed that there was no significant reduction in RNA levels in pizza or mincemeat after freezing and storage at −18 °C for 14 days. NoV outbreaks have been associated with ice (Cannon et al. 1991; Khan et al. 1994; Levine et al. 1990), and in recent years, have been particularly problematic after the consumption of frozen raspberries (Cotterelle et al. 2005; Falkenhorst et al. 2005; Sarvikivi et al. 2011). Therefore, frozen products are common vectors of human NoV illness.

No studies have been performed to evaluate the persistence of GII.4 NoV after repeated freeze–thaw (F/T) cycles; therefore, the effects of freezing and thawing on NoV, as would be encountered under certain environmental conditions, are unclear. It is possible that NoV in lakes, streams, and coastal areas may be affected by freezing and thawing although a strikingly higher incidence of NoV illness occurs during the winter months and is what led to the earlier name for NoV illness as “winter vomiting disease” (McSwiggan et al. 1978). Increased winter outbreaks may be associated with enhanced virus persistence in cold climates, less sunshine and ultraviolet irradiation to inactivate virus particles during the winter, or a generally lower resistance to illness among the population at large.

Some laboratory conditions under which NoV-containing samples are stored or preserved involve freezing and thawing even though freezing and thawing might be expected to compromise capsid integrity. Uncertainty about the effects of freezing and thawing on NoV capsid integrity has led to differences in the way laboratories preserve virus-containing specimens. For instance, NoV-containing stools from outbreak investigations are maintained in the refrigerator at many testing laboratories, pending analysis. Specimens are presumed to last for extended periods under these conditions although mold can overgrow the specimens. Other laboratories maintain specimens frozen until tested. For NoV analysis by RT-PCR, samples containing NoV are often aliquoted, frozen, and thawed as few times as possible, and used either as test samples, research materials, or positive controls. The need to maintain viral RNA titers in the laboratory is of paramount importance so further information is needed on the stability of NoV to freezing and to repeated cycles of freezing and thawing. If capsids lose their integrity from freezing and thawing or repeated freezing and thawing, then RNA titers, as determined by molecular methods, would decrease.

In this paper, we demonstrate that single or repeated cycles of freezing and thawing of diluted NoV stool stocks have no demonstrable effect on NoV titers even after RNase treatment. In addition, we show that NoV stool dilutions maintain their ability to bind to porcine gastric mucin, a potential proxy measurement for infectivity, after repeated F/T cycles.

## Materials and Methods

### Norovirus Stool

Fresh stools were provided to the Delaware Division of Public Health Laboratory for routine norovirus screening after outbreaks of gastroenteritis in long-term care facilities. All stools were sequence confirmed to contain GII.4 NoV (New Orleans) and the high-titered stools were reserved at 4 °C for this study. Stools used in this study showed high titer by end-point dilution RT-PCR (≥1 × 10^9^ RT-PCR units/ml) as determined by the method described in Richards et al. (2004). For NoV stock preparation and titration, stools were diluted 10-fold in sterile, nuclease-free water, vortex mixed, centrifuged at 10,000×*g* for 10 min, and serially filtered through sterile Millex-HV 0.45- and Millex-GV 0.22-μm Teflon, low protein binding syringe filters (Millipore, Billerica, MA, USA). These NoV “filtered stocks” were maintained at 4 °C until use.

### Freeze/Thaw

For each of two experiments, fourteen 500-μl tubes were each inoculated with 225 μl of filtered NoV stock and placed in a −80 °C freezer. Initially, samples were stored for up to 120 days at −80 °C with weekly or bi-weekly analysis after a single, slow (30 min) thaw at room temperature (about 22 °C). Thawing of all tubes was conducted at room temperature in styrofoam racks to insure slow thawing and to maximize damage of viral capsids from ice crystals. Complete thawing was achieved within 30 min. The RNA was extracted from samples, and the extracts were tested in triplicate by real-time RT-PCR, as described below.

A second set of 14 tubes was repetitively frozen at −80 °C and slowly thawed at room temperature at weekly intervals over the course of 14 weeks such that tubes underwent from 1–14 F/T cycles. At the designated weekly interval, the repeatedly frozen and thawed samples were thawed as a group. Tubes were transferred to another rack for storage at −80 °C when their designated number of F/T cycles was reached. Remaining tubes from that group were refrozen at −80 °C to await additional F/T cycles. Thawing was performed for 30 min for each time interval. One final thaw was performed on all samples at the end of the study and samples were subjected to viral RNA extraction and analysis by RT-PCR.

### Viral RNA Extraction

Using 140 μl of stool filtrate, NoV RNA was extracted, column purified, and eluted in 60 μl of water using a Qiamp Viral RNA Mini Kit (Qiagen Inc., Valencia, CA, USA) according to the manufacturer’s instructions. Positive controls consisted of similar NoV RNA extracts from the same stool filtrates, except samples were never frozen. For samples frozen and thawed, 140 μl were similarly extracted, concentrated to 60 μl, and assayed by real-time RT-PCR.

### Norovirus Real-Time RT-PCR

Real-time RT-PCR was performed on a Smart Cycler (Cepheid, Sunnyvale, CA, USA) using the one step RT-PCR Kit (Qiagen). Primers were as follows: forward primer QNIF2 (5′-ATGTTCAGRTGGATGAGRTTCTCWGA-3′) (Loisy et al. 2005), where R = A or G and W = A or T; reverse primer COG2R (5′-TCGACGCCATCTTCATTCACA-3′) (Kageyama et al. 2003). The probe was as described by Loisy et al. (2005), designated QN1FS, but Texas Red was substituted on the 5′ end and Black Hole Quencher 2 (BHQ-2) was on the 3′ end as follows: 5′-TexRed-AGCACGTGGGAGGGGATCG-BHQ-2-3′. Primers and probe were synthesized by Integrated DNA Technologies (Skokie, IL, USA). Final RT-PCR concentrations were 250 nM probe, 500 nM forward primer, 900 nM reverse primer, 5 μl buffer, 1 μl dNTPs, 1 μl enzyme mix, 5 U of RNase inhibitor (Invitrogen, Carlsbad, CA, USA), 12.25 μl nuclease-free H_2_O, and 1 μl viral RNA extract (template). Reverse transcription was performed at 50 °C for 30 min, followed by Taq activation for 15 min at 95 °C and then 50 cycles of PCR amplification at 95 °C for 15 s and 60 °C for 1 min. Negative controls consisted of master mix including primers with water substituted for the NoV stool extracts. Positive controls were as described above. Norovirus RNA titers of the diluted stool filtrates were monitored by real-time RT-PCR. Cycle threshold (C*t*) values were measured at an arbitrary threshold of 30 fluorescence units at the end of each 60 °C extension step. All aliquots were compared among each other for relative C*t* values. All extracts were tested by real-time RT-PCR in triplicate.

### RNase Treatments

The integrity of the NoV capsid to repeated (1–14) F/T cycles was monitored after the addition of exogenous RNase (RNace-it Ribonuclease Cocktail, Stratagene, La Jolla, CA, USA), which contained 2 μg/μl of RNase A and 4 U/μl of nonspecific RNase from *Aspergillus oryzae*. In this study, 150 μl of filtered NoV stock was added to each of 15-0.5 ml tubes. A non-frozen control was maintained in the refrigerator while the remaining tubes were frozen at −80 °C for ≥2 h. Thawing was performed at room temperature for 30 min in a styrofoam rack. At the end of the appropriate number of F/T cycles, all tubes were thawed and treated with 2 μl of RNace-it Ribonuclease cocktail to digest any viral RNA exposed by the freezing and thawing. Each tube was mixed by gentle vortexing, spun 2 s, and incubated at room temperature for 30 min. RNase inhibitor was added (5 μl = 50 U) to each tube to stop the reaction, and the samples were again mixed and spun for 2 s. After 5 min, the tubes were added to ice pending RNA extraction by Qiamp Viral RNA Mini Kit (Qiagen) and assay by RT-PCR. This trial was performed in duplicate and each extract was tested by RT-PCR in duplicate (*n* = 4). Previously, this RNase treatment was demonstrated to effectively eliminate all traces of NoV RNA in RNA extracts derived from the filtered NoV stock.

### Mucin-Binding Study

Fourteen 0.5-ml tubes were inoculated with 150 μl of filtered NoV stock. Tubes were frozen at −80 °C and slowly thawed at room temperature 1–14×, as described above. Porcine gastric mucin-conjugated magnetic beads (PGM-MBs) were prepared as previously described (Dancho et al. 2012). Binding studies were performed according to the procedures of Dancho et al. (2012) with the exception that a saturation titration curve was not included. Accordingly, infectious NoVs have greater binding affinities toward mucin-conjugated beads than non-infectious capsids (Dancho et al. 2012). Fifty microliters from each tube was diluted 1:20 by addition to 950 μl of PBS in duplicate. Fifty microliters of PGM-MBs were added to the 950 μl of diluted sample followed by rotational mixing at 8 rpm at room temperature for 30 min. A magnetic bead attractor (Stratagene) was used to separate the PGM-bound NoV from the unbound. The unbound viruses in the aqueous phase were removed from the bead extractor and kept on ice, while the viruses bound to the beads were washed 3× with 1 ml PBS containing 10 U of RNase inhibitor (Invitrogen). Heat release and concentration of the virus particles from the beads was accomplished by the addition of 50 μl of PBS followed by heating at 99 °C for 5 min. The magnetic beads were removed from the heat-released viruses using the magnetic bead attractor. Samples were placed on ice pending RT-PCR of the bound and unbound fractions. The trial was performed in duplicate for each sample and relative quantification of extracted RNA was based on observed C*t* values by real-time RT-PCR, which was performed in duplicate.

### Virus Quantification

In preliminary testing, it was determined that there was little to no change in the levels of NoV amplicon after 14 FT cycles; therefore, C*t* values were judged a good measure for relative changes in titers over the course of this study. Significant differences in NoV RNA levels were calculated by Student’s *t*-tests.

## Results

### Norovirus-Filtered Stock

The titers of the 1:10 diluted and filtered GII.4 NoV stocks used throughout this study were ≥1 × 10^8^ RT-PCR units/ml so the titers of the original, undiluted stools were ≥1 × 10^9^ RT-PCR units/ml. For each 10-fold dilution of the filtered NoV stocks, the C*t* value increased by a mean of 3.5 C*t* units.

### Single Versus Multiple Freeze Thaws

**Fig. 1:**
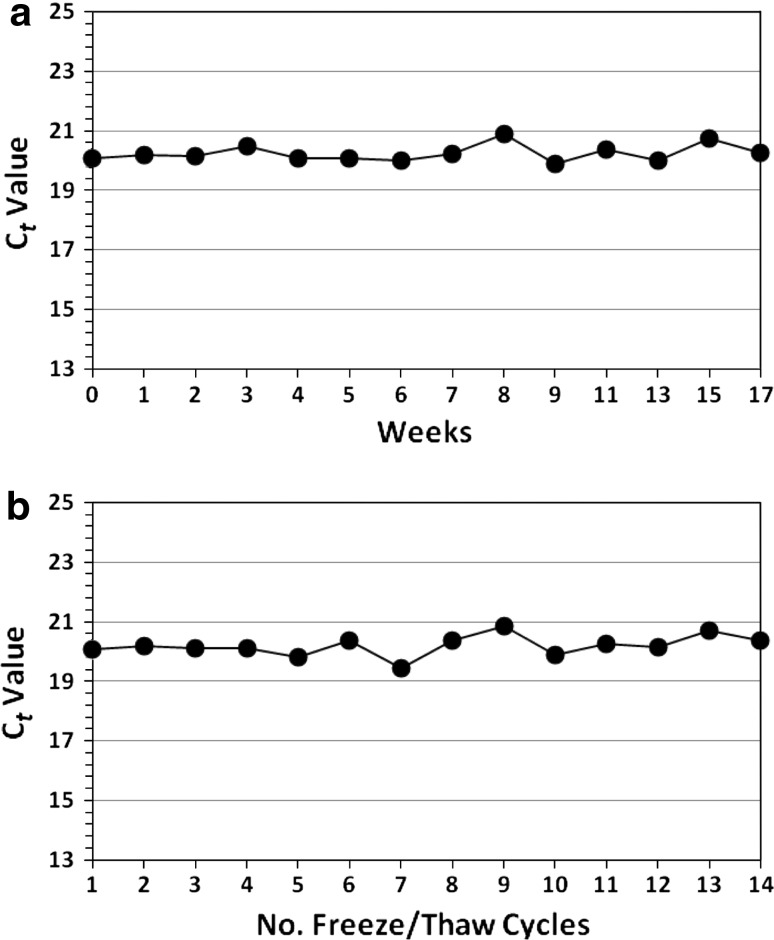
Relative cycle threshold (C*t*) values for GII.4 norovirus in water after **a** frozen storage and a single thaw weekly or every two weeks (biweekly) for up to 17 weeks (120 days) and **b** frozen storage and up to 14 freeze–thaw cycles at weekly or biweekly intervals. SD *bars* are too small to be seen

The effect of storage duration at −80 °C was evaluated using an initial freeze and then a single, room temperature thaw at weekly or bi-weekly intervals for 17 weeks (120 days), followed by immediate viral RNA extraction and real-time RT-PCR analysis. The relative viral RNA titers of the extracts remained essentially the same over the 17-week trial period (Fig. 1a). For the multiple freeze/thaw experiment, aliquots were frozen and thawed up to 14×. Relative viral RNA levels remained essentially the same, as judged by relative C*t* values (Fig. 1b) for those thawed 1–14×. The mean C*t* values for the aliquots frozen and thawed only once were not significantly different from the values derived from aliquots which were repeatedly frozen and thawed (Fig. 1a, b respectively). The precision of the results was high given the very small standard deviation bars in Fig. 1 (mentioned as too small to be seen in the figure legend) and later in Fig. 3 (where only some were large enough to be visualized). Overall, there was no reduction in NoV titer observed for either experiment; the mean titer of the initial sample before freezing (C*t* = 20.05) was not significantly different (*P* > 0.05) from the mean titers of the samples frozen once (C*t* = 20.25) or up to 14× (C*t* = 20.36).

### Retention of Capsid Integrity

**Fig. 2:**
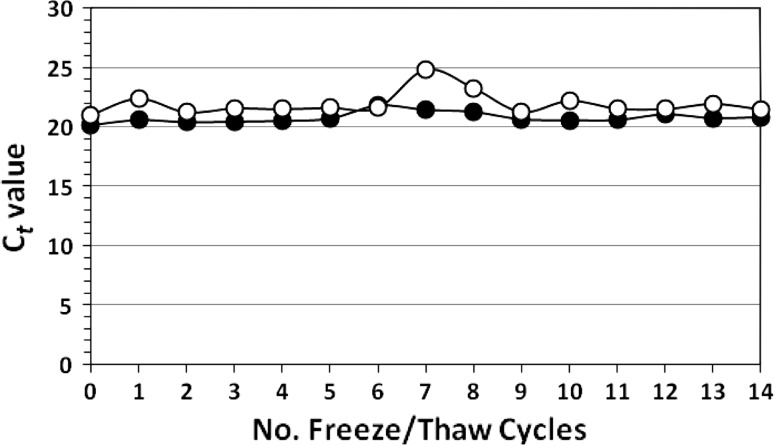
Two independent trials showing GII.4 norovirus persistence in water after 0–14 freeze/thaw cycles followed by the addition of RNase cocktail. Relative cycle threshold (C*t*) values were determined in triplicate for each trial

To confirm that repeated F/T cycles do not release the viral RNA from the virion, RNase was added to assess whether the capsid was capable of protecting the viral genome from enzymatic destruction. Norovirus was frozen (−80 °C) and slowly thawed (+22 °C) from 1–14× followed by the addition of commercially obtained RNase cocktail after the designated number of F/T cycles. After incubation for 30 min, RNase inhibitor was added and viral RNA was extracted and assayed. Unfrozen NoV stock filtrate served as a control. Results confirm no loss of NoV RNA titers over 14 F/T cycles (Fig. 2). The initial, unfrozen titer (0 F/T) was not significantly different (*P* > 0.05) from the titer after 14 F/T cycles (mean C*t* values of 20.6 and 21.2, respectively). Some increases in C*t* values (suggesting about a 1-log and 0.5-log decrease in titer) were observed at 7 and 8 F/T cycles in one of the trials, but are attributed to the variability in column extraction of the NoV RNA since C*t* values remained low and nearly constant from 2 to 6 and 9–14 F/T cycles (Fig. 2).

### Retention of Capsid Function

**Fig. 3:**
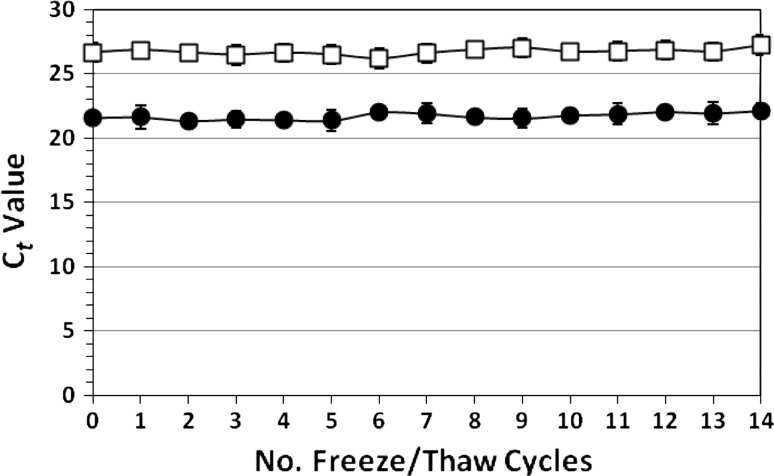
Binding of frozen and thawed GII.4 norovirus to porcine mucin. Relative cycle threshold (C*t*) values for porcine mucin-bound (*filled circle*) and unbound (*empty square*) norovirus, where binding may be a predictor of norovirus infectivity. *Bars* indicate SD

A recently developed porcine gastric mucin binding assay, which measures the ability of the intact capsid to bind to receptor-like glycoproteins was employed as an indicator of potential NoV infectivity. Bound and unbound NoVs showed no significant change (*P* > 0.05) in binding efficiency over 14 F/T cycles (Fig. 3). The mean C*t* value for the bound and unfrozen control was 21.6 compared with the bound sample which was frozen and thawed 14× and which gave a C*t* of 22.1. The mean C*t* values for the unbound, unfrozen control and for the unbound sample that had been frozen and thawed 14× were 26.7 and 27.2, respectively. In total, the data presented here suggest that neither the integrity nor the infectivity of GII.4 NoV in an aqueous stool suspension was significantly affected by up to 14 F/T cycles.

## Discussion

To our knowledge, this is the first time that the effects of multiple F/T cycles on human NoV have been evaluated and results conclude that freezing and thawing does not affect capsid integrity or the RNA titer. Evidence that NoV RNA remains encapsidated after freezing and thawing is supported by the fact that unfrozen samples and samples frozen and thawed only once over the course of 17 weeks (120 days) contained essentially the same levels of viral RNA as samples frozen and thawed up to 14×. Freezing followed by slow thawing at room temperature would be expected to exert maximum damage to viral capsids from prolonged exposure to ice crystals. If F/T damage had substantially affected the capsid’s integrity, then the viral RNA would have been destroyed by ubiquitous RNases present in stool filtrates. These RNases would include a variety of endo- and exonucleases from both human and bacterial cells in the gut. While RNases are considered very hardy enzymes, it is conceivable that RNases from the stool filtrates could be damaged and inactivated after repeated F/T cycles. Consequently, we performed additional tests where a commercially obtained RNase cocktail was added to NoV after 1–14 F/T cycles. It showed that there was also no reduction in virus titer after repeated freezing and thawing.

Another line of evidence for capsid integrity is predicated on the ability of viable viruses to bind to porcine gastric mucin. It is well established that the detection of viral RNA does not indicate the presence of infectious viruses, because virus infectivity also depends on the retention of capsid protein conformation (Richards 1999). For a NoV to initiate an infection, the capsid must mediate binding of the virion to a host cell via a receptor and the virion must subsequently enter the cell and release the positive-sense RNA genome into the cytosol. Essentially, the porcine mucin binding assay is a proxy for the first step in the replication process (e.g., receptor binding). Previous work has shown that NoV subjected to thermal, UV, and high pressure treatments retain capsid integrity; however, the treatments damage the capsid and prevent capsid binding to mucin (Dancho et al. 2012). In this paper, we have extended earlier work to show that repeated freezing and thawing does not affect the capsid’s ability to bind to mucin, suggesting that multiple F/T cycles do not reduce NoV infectivity. The absolute confirmation of virus infectivity would require an assessment using human volunteers.

Overall, we conclude that NoV GII.4 is highly resistant to repeated F/T cycles and to long-term storage. It is uncertain if other genogroups or clades of NoV offer this same cryostability. Our results, showing the persistence of NoV to long-term freezing, are consistent with a NoV volunteer study which demonstrated that GI.1 NoV (Norwalk strain 8FIIa) retained infectivity after frozen storage for more than two decades (Teunis et al. 2008). In contrast, a study using the propagatable NoV surrogate, feline calicivirus, showed some virus sensitivity to repeated F/T cycles (Duzier et al. 2004), suggesting that this surrogate may not be representative of human NoV. One should be careful in using surrogate data to predict a response for human NoV since surrogates often do not mimic the viruses they represent (Richards 2012).

The persistence of NoV to freezing and thawing is unwelcome news in food processing and for the elimination of NoV from the environment. For foods potentially contaminated with NoV, such as produce and shellfish, there is no evidence to suggest that freezing and thawing will have a beneficial effect on reducing virus infectivity. In fact, food matrix and proteins would be expected to further stabilize NoV over water alone. Thus, we conclude that freezing and thawing are unsuitable as a food processing intervention for NoV. Likewise, our results indicate that winter freezing and thawing events are not likely to contribute to the inactivation of environmental NoVs. However, this persistence of NoV to freezing and thawing is welcome news in the laboratory, where samples destined for analysis or to be used as positive controls may be repeatedly frozen and thawed without any anticipated reduction in viral RNA titers.
